# Free-Living Amoebas in Extreme Environments: The True Survival in our Planet

**DOI:** 10.1155/2022/2359883

**Published:** 2022-10-18

**Authors:** Camila Salazar-Ardiles, Leyla Asserella-Rebollo, David C. Andrade

**Affiliations:** ^1^Research Center in High Altitude Medicine and Physiology, Biomedical Department, Faculty of Health Science, University of Antofagasta, Antofagasta, Chile; ^2^Medical Technology School, Faculty of Health, University Santo Tomás, Chile

## Abstract

Free-living amoebas (FLAs) are microorganisms, unicellular protozoa widely distributed in nature and present in different environments, such as water or soil; they are maintained in ecosystems and play a fundamental role in the biological control of bacteria, other protozoa, and mushrooms. In particular circumstances, some can reach humans or animals, promoting several health complications. Notably, FLAs are characterized by a robust capacity to survive in extreme environments. However, currently, there is no updated information on the existence and distribution of this protozoan in inhospitable places. Undoubtedly, the cellular physiology of these protozoan microorganisms is very particular. They can resist and live in extreme environments due to their encysting capacity and tolerance to different osmolarities, temperatures, and other environmental factors, which give them excellent adaptative resistance. In this review, we summarized the most relevant evidence related to FLAs and the possible mechanism, which could explain their adaptative capacity to several extreme environments.

## 1. Introduction

Amoebas are unicellular protist microorganisms belonging to the genus amoeba of the family Amoebidae that contains five described genera. Free-living amoebas (FLAs) develop their lives in the environment and are characterized by the lack of a cell wall in the trophozoite stage, which allows them to extend their cytoplasm to mobilize, resulting in the formation of pseudopods, further enabling them to feed on smaller microorganisms, mainly bacteria or decaying particles. Therefore, it plays an essential biological role in the control of bacterial populations. [1, 2]. Amoebas are found in various environments; however, environmental conditions can affect their survival, such as pH, temperature, hydrogen sulfide concentration, and salinity. These conditions directly influence the structure of amoeba communities, mainly in aquatic-type environments [3]. These microorganisms survive in adverse environments using osmoregulation to control the water inside to cell through vacuoles [4]. This mechanism is regulated by the expression of aquaporins in the contractile vacuole membrane. The expression of V-ATPase (vacuolar H^+^-ATPase) in the vacuolar membrane has been reported. V-ATPases move H^+^ inside, lowering the vacuole's pH relative to the cytosol, promoting an osmosis gradient across the vacuole membrane, facilitating water entry through aquaporins in the vacuole membrane [5, 6].

The first description of an amoeba was in 1826 as a membranous type of microorganism whose shape is modified to move in the environment [7]. However, the first published evidence of this microorganism dates back to 1755 by the German naturist Rösel von Rosenhof AJ in Insecten-Belustigung (extracted from [8]). Currently, there are two classes of amoebae that differ from each other; FLA are those that live free in the environment that do not strictly need a host, and parasitic that needs a host and are mostly pathogenic to humans with the ability to generate serious diseases [8].

Until 2002, amoebas, such as eukaryotic microorganisms, have been studied mainly for their morphology, characterizing them as testate amoebas; however, few differences and morphological structures led to the lack of classification and differentiation between the diverse species of amoebas [9]. With the advances in molecular biology, a new classification era of these organisms arises, with the development of sequencing of specific genes based initially on a small sequence of the 18 s gene, or SSU-rDNA [10]. After, universal and conserved genes were used (cytoskeleton actin and tubulin protein genes) that have brought with them greater resolution in the identification and classification of supergroups [11]. Accordingly, three supergroups that have been identified were, Amoebozoa supergroup brings together most of the organisms capable of producing lobed pseudopods, Rhizaria, which is characterized by creating filamentous pseudopods; and the Excavata, which brings together a large part of the flagellate heterotrophs [11, 12].

With all current information, it is possible to speculate that the differences in amoebas could be associated with the presence or absence of flagella, developed with the expansion of these organisms to different substrates to seek favorable conditions promoting their survival, searching for nutrients from bacteria. The evolution of these microorganisms is not entirely clear; however, fossil evidence of Arcellinida testate proves the first appearance of eukaryotic cells for more than 700 million years [13, 14].

As aforementioned, these cosmopolitan organisms can be found across environments where life is carried to the maximum of its survival capabilities (extreme environments), with extremely high or low ranges of temperature, radiation, pressure, acidity, alkalinity, salinity, sulfur, and among others. FLAs, similar to other species, have developed several strategies to survive in hostile environments through the formation of cysts [15]. Indeed, cyst-forming FLAs can survive in several environments for many years. Thus, it has been shown that survival can range between 2 and 21 years, even in arid and cold climates (~4°C), offering high viability, particularly those belonging to the genus *Acanthamoeba* [16–18]. Indeed, through their robust adaptability [19, 20], FLAs could play an essential epidemiological role as a reservoir and vehicle for a wide variety of microorganisms; in addition, they contribute to plant growth, soil mineralization, and nutrient cycling [21]. Of note, it has been attributed to the genera *Acanthamoeba* spp., *Naegleria fowleri*, *Balamuthia mandrillaris*, *Sappinia pedata*, *Vermamoeba vermiformis*, and *Paravahlkamfia francinae* (all free-living amoebas) as etiologic factors in fatal central nervous system infections and other human serious diseases [8, 22–24]. These are amphizoid amoebas because they live as parasites on hosts and in their natural environment [25, 26].

Antibodies against *Acanthamoeba* are present in approximately 80% of the human population [27], suggesting that this FLA is an organism that comes into contact with humans regularly [28]. *Acanthamoeba* species do not require a host to survive; they can settle in tissues and cause serious illness [29–31]. Some species belonging to the genera *Acanthamoebae*, *Naegleria*, *Balamuthia mandrillaris,* and *Sappinia pedata* are carriers of other pathogens such as *Legionella pneumophilia*, *Listeria monocytogenes*, *Mycobacterium avium*, *Pseudomonas aeruginosa*, *Pseudomona saccharophilia*, and among others [32–36]. In addition to being associated with human diseases, these amoebas play an essential role in ecosystems, acting as predators and hosts of microorganisms such as *Cryptosporidium* and *Toxoplasma* [37–39]. Thus, the present review is focused on summaries of the more critical evidence related to amoebas living in extreme environments, and we will propose some hypothetical mechanisms which could help to explain, in part, this characteristic of the amoebas residing in harsh environments for several time. Accordingly, we could arise how these organisms survived for so many years on the earth.

### 1.1. Free-Living Amoebae

**Figure 1:**
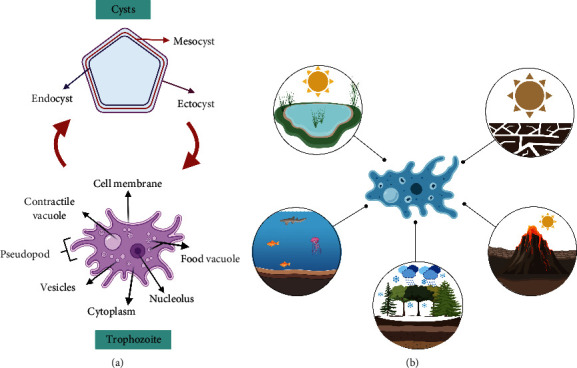
Free-living amoeba life cycle diagram. (a) Structure of the cyst, composed of three-layer: endocyst, mesocyst, and ectocyst. The presence of layer mesocyst is dependent on the genus. The trophozoite is the metabolically active vegetative form. (b) Diagram of environments where FLA can be found, created with http://BioRender.com/.

FLAs maintain their life cycle in the environment. Still, some species act as opportunistic and nonopportunistic pathogens that can affect different hosts, such as animals and humans, and thus carry out a monoxene life cycle. *Acanthamoeba* and *Naegleria* are referred to as amphizoid organisms because they can exist as both FLA and pathogenic parasites (Centers for Disease Control and Prevention [40]. Amoebas of the genus *Acanthamoeba* present two stages during their life cycle: (a) trophozoite (13–40 *μ*m in diameter) or metabolically active vegetative form, which feeds on bacteria and smaller organisms and multiplies by binary fission, giving rise to two identical daughter cells and (b) cysts (6–10 *μ*m in diameter) or forms of resistance [41–44]. Other amoebas, such as those in the genus *Naegleria*, have three morphological stages, one of which is a temporary amoeboflagellar stage in which the organism does not feed or reproduce and only serves to move to a better microenvironment [1, 45]. The cysts originate from the production of a protective covering by the trophozoite when it is under extreme environmental conditions such as changes in temperature, humidity, pH, nutrients, osmotic pressure, and among others, which covers it and turns it into a form of resistance. It generally has two layers: an external or ectocyst and an internal or endocyst; a third layer, the mesocyst, is present in some species. These cystic structures may explain why the cyst is so resistant to various disinfection methods, such as chlorination and sterilization of water systems, and mainly how they can survive extreme environments, such as the lagoons of the Atacama Desert see Figure 1. Inside the cyst, the trophozoite remains inactive until it is in a favorable environment, which initiates the process of disembedding [8]. Thus, the evidence is robust to show the resistance of the amoeba to survival in extreme environments, characterized by changes in humidity, temperature, and nutrients, which could affect its activity, quantity, and diversity [8].

### 1.2. Taxonomy of Free-Living Amoebas

The International Society of Protozoologists proposed a new classification based on morphological, biochemical, and phylogenetic approaches. Eukaryotes are classified into six supergroups: *Amoebozoa, Opisthokonta, Rhizaria, Archaeplastida, Chromalveota,* and *Excavata* [46]. Three of these six groups correspond to FLA groups, such as the *Amoebozoa* supergroup, which has noneruptive pseudopods called lobopods that can be branched. The gymnamoebas, naked amoebas and a significant number of testate amoebas are grouped here. The *Rhizaria* supergroup amoebae have very fine pseudopods that can be simple, branched, anastomosing, or microtubule support (axopods). They have a very diverse way of life; some species are photosynthetic or parasites of plants and other organisms; they are fresh and saltwater aquatic amoebas, but they can also be found in soils [46, 47]. In many cases, a group of amoebae in the *Excavata* supergroup can have a flagellated phase of their life cycle. Although they differ from amebozoans, eruptive pseudopodia predominate in these organisms [1]. *Acanthamoeba* and *Balamuthia* are members of the *Acanthamoebidae* family, which is part of the *Amoebozoa* supergroup. The *Excavata* supergroup of the *Vahlkampfiidae* family includes *Naegleria fowleri*, *Vahlkampfia*, and *Willaertia Sappinia*, a family member of *Thecamoebidae* in the supergroup *Amoebozoa* [46, 48, 49].

**Figure 2:**
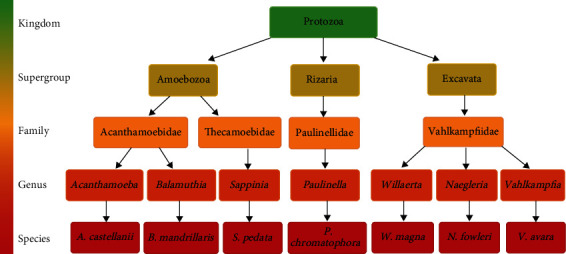
Taxonomic classification of free-living amoebas. The figure shows the taxonomic classification of the species corresponding to free-living amoebas. The figure was made based on the following authors [45, 47, 106–108]), created with http://BioRender.com/.

Fowler and Carter discovered the *Acanthamoeba* and *Naegleria* genera in 1965, respectively. Some *Acanthamoeba* species were initially divided into three morphological groups (I, II, and III) [50, 51]. However, at the *Acanthamoeba* species level, this morphological classification is incorrect [52, 53]. The genus taxonomy is primarily based on morphological characteristics such as cyst size and morphology [19]. The disadvantage of this type of diagnosis based on morphological characteristics of the cyst varies depending on the method used [36]. Therefore, it is necessary to use cultures and molecular identification from specific primers for correct identification [43, 53]. Commonly used primers are based on the amplification of the particular genes for both species and genera of amoebae, such as FLA, which corresponds to a universal primer of the 18S rDNA gene [54], JDP, BAL, ITS, and NFITSFW name the specific primers for the genus *Acanthamoeba* spp. [54], *Balamuthia mandrillaris* [55], *Naegleria* spp. [56], and *Naegleria fowleri* [57, 58], respectively. In addition, it has been proposed that FLA diversity could be associated with developed places [59] it is believed such extreme environments, which could confer different properties to the FLA, a summary of the FLA taxonomy is shown in Figure 2.

Currently, 22 genotypes for the genus *Acanthamoeba* have been identified, ranging from T1 to T22, based on slight differences detected in regions of the rDNA gene, of which less than 5% are recognized as a single genotype [53, 60–64].

### 1.3. Pathogenicity Promoted by Free-Living Amoebas and Possible Treatment

It has been demonstrated that FLAs can promote several serious diseases. Among the most abundant amoebas in nature are the *Acanthamoeba* and signalment *B*. *mandrillaris*, which have been recognized as opportunistic human pathogens enabling blindness by *Acanthamoeba* keratitis and rare but fatal granulomatous encephalitis (GAE) by signalment *B*. *mandrillaris* [24, 65]. *Naegleria fowleri* is another pathogen FLA, known to cause central nervous system infection and primary amoebic meningoencephalitis, a pathophysiological state with a poor survival rate [24]. Of note, FLAs are not only recognized as disease causing, if not, which also act as vehicles for pathogenic bacteria [66]. All this evidence depicted the relevant role of FLA in promoting several human pathophysiological conditions.

Although it is very well known that FLAs can promote several serious diseases, most medical solutions have been effectively used. Indeed, current treatments combining drugs, such as amphotericin B and miltefosine, have been to be effective in treating Primary Amoebic Meningoencephalitis (PAM) symptoms disease, caused by *Naegleria fowleri*; however, without reducing the number of deaths (mortality rate of 95%) [67]. In addition, there are amebicidal agents, among which, both diamidines and biguanides display the capacity to destroy cystic forms. Diamidines, propamidine isethionate, and hexamidine are all agents that have been disclosed for the management of *Acanthamoeba* keratitis or neomycin when signs of corneal toxicity were present [68]. Among the biguanides, polyhexamethyl biguanide (PHMB), generally used at 0.02%, and chlorhexidine at 0.02% stand out [69, 70]. These drugs were used when patients had toxicity problems with diamidines.

There are other groups of medications that are not used as first-line drugs as they present toxic effects on the corneal epithelium when used for prolonged periods; these are the aminoglycosides (neomycin) and the imidazole [71, 72]. Other treatment schemes reported with success rates are propamidine with 1% miconazole, propamidine with topical 1 or 2% clotrimazole, and 0.1% miconazole plus debridement with itraconazole or oral ketoconazole. All these regimens were accompanied by neomycin polymyxin B sulfate.

Treatments usually used with positive results for corneal infections by *Acanthamoeba* (corneal ulcers), a combination of 0.02% chlorhexidine is used topically every two hours and topical polymyxin B neomycin every six hours, added to ketoconazole 200 mg orally every 12 hours. A follow-up is performed at two weeks to see the evolution of the disease and to continue until six weeks exclusively with 0.02% topical chlorhexidine every two hours. However, they are slow growing but effective with long-term treatment [69, 73]). However, although apparently, the treatments could be effective in treating amoeba infection, recently, have been tested new treatments options to improve the response of the immune system, but has been only tested in preclinical models with promising results [74, 75].

### 1.4. Identification of Amoebae in Extreme Environments

FLA has been isolated in extreme environments, such as the desert, at different soil depths and times of the year. Soil characteristics differ in terms of humidity and organic matter availability. Of note, a relationship has been observed between amoeba abundance and the number of bacteria present in the soil [76, 77]. Regarding extreme environments, Rodriguez-Zaragosa et al. isolated amoebas from the Negev Desert in Israel, including *Acanthamoeba*, *Vahlkampfia*, *Naegleria*, *Willaertia*, *Tetramitus*, *Paratetramitus*, and *Adelphamoeba*. This same group isolated 57 strains from desert environments, of which 39% belonged to the genus *Acanthamoeba* (which is essential to human health because it causes amoebic keratitis and granulomatous encephalitis), 16% to *Hartmannella*, 9% to *Vahlkampfia*, and the remaining proportion was divided among six other genera. Another factor, such as a decrease in bacterial load during the winter, increases the presence of flagellated amoebas because they can move freely to reach the microorganisms and so be able to feed. All these findings demonstrate the remarkable adaptability of FLA to survive in different extreme environments, and a new question arises about the mechanism of FLA and how it responds to other abiotic, biotic, and predatory prey [29, 78–81].

One of the more adaptative FLA is the *Acanthamoeba*, one of nature's most abundant genera, having been isolated from a wide range of environments, including freshwater pools and desert soil samples. Similarly, *Balamuthia mandrillaris* has been found in several environments, including hot tropical climates and cold regions with heavy snowfalls in northern Japan, where it was discovered for the first time in this type of cold environment [82]. In addition, regarding temperature, the genus *Willaertia magna* is a thermophilic FLA with an optimal growth temperature of 43°C. In its vegetative form named trophozoic, it measures between 50 to 100 *μ*m in diameter and around 18 to 21 *μ*m when it is cystic [83]. The *W. magna* is neither pathogenic, toxic nor ecotoxic. Different strains have been isolated from different extreme environments, such as Z503 and Z504, which were isolated from feces taken directly from the bovine rectum; TS-9 from the soil in France, M-1 from samples of contaminated thermal water in France, and strain T5 (S) 44 of sediment in the thermal effluent of a nuclear power plant in Belgium [84, 85]. In addition, the genus *Acanthamoeba castellanii* has been found in samples taken from frozen waters for recreational use in Oslo and Norway, demonstrating the wide distribution and resistance of the genus [86].

Astorga reported the presence of *Acanthamoeba* spp. in all regions of Chile and during all seasons of the year (from the region I to region XV) using phenotypic and genotypic identification. Water from Easter Island's Rano Kao crater and thermal waters obtained throughout Chile were analyzed, yielding temperature records of 44°C and declaring the thermotolerance; the distribution found was of *Acanthamoeba* spp 38.4%, *Varmamoeba vermiformis* 53.8%, *Naegleria* spp 12.8%, *Stenamoeba* spp 15.4%, *Filamoeba* spp 5.1%, and *Heterolobosea* 2.6%.

*Acanthamoeba* grows in water, soil, and plant samples at average temperatures such as 42°C. Groups II (84%) and III (84%) contained isolated *Acanthamoeba* spp. The T4 genotype predominates in Group II, and the T5 genotype predominates in Group III, genotypes that rank first and second in environmental studies; thus, all are potentially pathogenic, according to the literature. Isolated *Acanthamoeba* spp. was found in all areas with very different characteristics in terms of climate, water availability, and soil type, demonstrating its ubiquity and potential risk and highlighting the predominance of the T4 genotype in waters, soils, and plants. The T4 genotype is mainly found in recreational waters. T2, T3, and T4 genotypes have been identified in nonrecreational environmental water samples [87]. Through morphological analysis and partial sequencing of 18S rDNA, researchers in Turkey discovered the presence of *Acanthamoeba* genotype T4 and *Vermamoeba vermiformis* in tap water samples [88].

Regarding geographical location, it has been shown that Khyber Pakhtunkhwa province, located in Pakistan, displays several health problems associated with the presence of pathogens, which have been related to access and quality of the water. In fact, through PCR, it was possible to identify amoebas of the genus *Acanthamoeba* using specific genes; sequencing results confirmed seven different pathogenic and nonpathogenic genotypes, including T2–T10, T4, T5, T7, T15, T16, and T17 [89]. In addition, new reports have shown the presence of *Acanthamoeba* spp. (65%), *Balamuthia mandrillaris* (5%), and *Naegleria fowleri* from water plants in Karachi City [90]. Rather worryingly, from drinking tap water was possible to identify *Acanthamoeba* spp. (35%), but without the presence of *Balamuthia mandrillaris* (5%) and *Naegleria fowleri* in this last type of sample [90].

### 1.5. Possible Mechanisms to Explain the Survival of FLA in Extreme Environment

FLA has displayed resistance to environmental adversities and germicides. However, some organisms have developed resistance to the intracellular milieu of amoebas, as in the case of *Acanthamoeba*, which has been functioning as excellent reservoirs for amoeba-resistant microorganisms (ARMs), such as bacteria, viruses, and fungi. Little is known about these relationships and interaction mechanisms. Still, it is speculated that the FLAs need a broad repertoire or universal class of receptors to bind and recognize these diverse species of microorganisms. Also, it has been demonstrated that the temperature, pH, concentration of sulfhydric acid, and salinity can affect the survival of FLA; these factors strongly influence the amoeba's structure [59, 91].

FLAs displayed resistance to adverse environments and germicides. Of note, amoebas have developed cysts in response to several stressor agents nearing favorable environmental conditions to return to their metabolic activity (trophozoite state). In addition, FLA serves as a reservoir to several microorganisms (bacterium, viruses, and fungi) which indeed have developed resistance to the intracellular milieu of amoebas, as in the case of *Acanthamoeba*, which has been acted as excellent carriers and reservoirs for amoeba-resistant microorganisms (ARMs). Notably, although a close relationship between FLA and ARM has been described, little is known about the interaction mechanisms. Still, it is speculated that FLA is being able to develop a vast repertoire or universal class of receptors to recognize and bind to these microorganisms. Notably, cyst formation in response to several stressor agents is one of the more important mechanisms of FLAs in extreme environments; however, the molecular mechanism of encystment is still to be elucidated. The cysts are double-walled and consist of ectocyst and endocyst. The ectocyst is formed during the initial stage of encystment. It appears to be an amorphous and discontinuous layer, while the endocyst has a fine granular texture and is uniformly thicker. In addition, ectocyst consists of a mixture of proteins and polysaccharides, and endocyst consists of polysaccharides, mainly cellulose [92]. *Acanthamoeba* cyst walls contain 33% proteins, 4–6% lipids, 35% carbohydrates (prominent cellulose), 8% ash, and 20% unidentified material [93]. The carbohydrate components of the cyst wall contained about 48% galactose and 44% glucose.

Furthermore, linkage analysis revealed the presence of 3-linked galactopyranose (1,3-linked galactose) as the highest constituent of the cyst wall (about 29%). At the same time, 4-linked glucopyranose (*β*-1,4-linked glucose, i.e., likely cellulose) was 22% as the second principal component [94]. Thus, since cyst formation is one of the primary mechanisms in response to extreme environments, it is possible to speculate that some cyst components could change in response to stressor agents. Indeed, it has been demonstrated that temperature, pH, the concentration of sulfhydryl acid, and salinity can affect the survival of FLA. These factors can strongly influence the structure of the amoeba, giving robust mechanisms to survive in several extreme environments [95].

**Figure 3:**
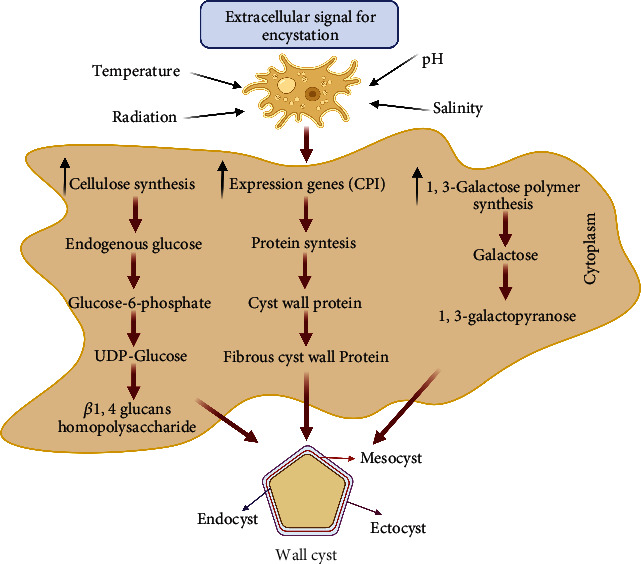
Mechanism cyst amoeba biosynthesis. The figure shows the formation encystment process mechanism of the cyst's wall. The formation of the cyst wall is characterized by three major components: protein synthesis, cellulose, and galactose polymers, which affect the amoeba wall formation, created with http://BioRender.com/.

Among carbohydrates, cellulose was identified as a significant constituent. The cellulose precursor is glucose incorporated into the cell wall as *β*(1 → 4)-glucans (i.e., cellulose). However, in *N*. *fowleri* expression studies, an increase in the expression of the cysteine protease inhibitor genes (belonging to the cystatin family) was evidenced in the encystment process and mature amoeba cysts in *N*. *fowleri* (NfCPI) [96]. These findings collectively suggest that NfCPI may play a critical role in *N*. *fowleri* cyst formation by regulating cysteine proteases that may be actively involved in mediating cyst formation or else in the encystment process of amoebae [97].  Figure 3 shows a summary of the cyst formation mechanism.

### 1.6. Comparison between Two Best-Known Species of Free-Living Amoebae

Considering that *Acanthamoeba* and *Naegleria* are the more common FLA on our planet and both promote several pathophysiological conditions are very interesting genera of amoebas to compare them. *Acanthamoeba* spp. is free-living naked microbial predator, disseminators of opportunistic infections, and can survive in extreme environments by forming double-layered cysts [98]. It is characterized by two stages, trophozoite (ameboid) and its resistance form (Cysts) [99]. *A*. *castillani* contains locomotor pseudopods, spikelike “acanthopodia,” and microprojections from the cell surface formed by hyaline cytoplasm excluding formed elements of the cell and containing a fine fibrillar material [100]. In contrast, *Naegleria fowleri* presents three stages, including a flagellar that allows it to move in aquatic environments in search of nutrients primarily from bacteria [101]. To date, more than 40 different species of *Naegleria* have been identified [99]. In contrast, *Acanthamoeba castellanii* consists of pathogenic and nonpathogenic strains and has been classified into 17 different genotypes from T1 to T17 [8, 102]. Some species of FLA may be involved in opportunistic and nonopportunistic infections in humans. Pathogenic FLAs belong to five genera, *Balamuthia*, *Acanthamoeba*, *Sappinia*, *Naegleria*, and *Vermamoeba* [99, 103]. Using a transcriptome approach, the flagellation process of *Naegleria* shows that it takes about one hour to be completed and requires the transcription of a set of the basal body and flagellar apparatus genes [104].

Regarding morphological characteristics*, Naegleria* cysts are spherical, 8 to 12 *μ*m in diameter, naturally resistant, and contain a single nucleus and a double wall with pores [105]. The cyst size of the *Acanthamoeba* can vary from 13 to 20 *μ*m depending on the species. In their resistance stages, they are transported through dust and can enter through the nose, where they begin the invasion [35].

### 1.7. Perspectives

All this evidence suggests that FLAs could be an excellent candidate as an experimental model to explain mechanisms related to extreme environmental survival. The evidence showed that FLAs could survive harsh environments, so they are of great interest to study biological processes such as osmoregulation and their molecular mechanisms.

In addition, there is an evidence of meaningful participation in the increase of infectious outbreaks of pathogenic bacteria, the development of antibiotic resistance (AMR), and the recombination of virulence genes between bacteria.

## 2. Conclusions

FLAs are microorganisms that can be found in several environments, such as aquatic or terrestrial, also in extreme environments, which can present different physical features that drive these microorganisms to develop different survival strategies. It has born a great interest in studying physiological mechanisms from the point of view of understanding how FLAs have developed strategies of adaptation that allow survival in several extreme environments. Understanding the molecular mechanism of these microorganisms, it could enable the development of biotechnological applications to be used in different fields of science, or they can also be used as biological models.

## Data Availability

No data were used to support this study.
